# Simultaneous Saccharification and Fermentation of Sugar Beet Pulp with Mixed Bacterial Cultures for Lactic Acid and Propylene Glycol Production

**DOI:** 10.3390/molecules21101380

**Published:** 2016-10-17

**Authors:** Joanna Berlowska, Weronika Cieciura, Sebastian Borowski, Marta Dudkiewicz, Michal Binczarski, Izabela Witonska, Anna Otlewska, Dorota Kregiel

**Affiliations:** 1 Institute of Fermentation Technology and Microbiology, Lodz University of Technology, Wolczanska 171/173, 90-924 Lodz, Poland; weronikacieciura@wp.pl (W.C.); sebastian.borowski@p.lodz.pl (S.B.); marta.dudkiewicz@p.lodz.pl (M.D.); anna.otlewska@p.lodz.pl (A.O.); dorota.kregiel@p.lodz.pl (D.K.); 2 Institute of General and Ecological Chemistry, Lodz University of Technology, Zeromskiego 116, 90-924 Lodz, Poland; michal.binczarski@p.lodz.pl (M.B.); izabela.witonska@p.lodz.pl (I.W.)

**Keywords:** sugar beet pulp, enzymatic hydrolysis, simultaneous fermentation, lactic acid, assimilation profile, *Lactobacillus*

## Abstract

Research into fermentative production of lactic acid from agricultural by-products has recently concentrated on the direct conversion of biomass, whereby pure sugars are replaced with inexpensive feedstock in the process of lactic acid production. In our studies, for the first time, the source of carbon used is sugar beet pulp, generated as a by-product of industrial sugar production. In this paper, we focus on the simultaneous saccharification of lignocellulosic biomass and fermentation of lactic acid, using mixed cultures with complementary assimilation profiles. Lactic acid is one of the primary platform chemicals, and can be used to synthesize a wide variety of useful products, including green propylene glycol. A series of controlled batch fermentations was conducted under various conditions, including pretreatment with enzymatic hydrolysis. Inoculation was performed in two sequential stages, to avoid carbon catabolite repression. Biologically-synthesized lactic acid was catalytically reduced to propylene glycol over 5% Ru/C. The highest lactic acid yield was obtained with mixed cultures. The yield of propylene glycol from the biological lactic acid was similar to that obtained with a water solution of pure lactic acid. Our results show that simultaneous saccharification and fermentation enables generation of lactic acid, suitable for further chemical transformations, from agricultural residues.

## 1. Introduction

Lactic acid (2-hydroxypropionic or 2-hydroxypropanoic) is the most widely produced acid using biotechnological methods [1]. This versatile carboxylic acid is used as an acidulant, flavoring, and preservative metabolite in the food, pharmaceutical, leather, and textile industries [2]. Research on the fermentative production of lactic acid from agricultural by-products has recently concentrated on the direct conversion of biomass. Inexpensive raw materials, such as starch or molasses, have been used to replace pure sugars in lactic acid production processes [3]. Lignocellulose, another carbohydrate source, is widely available and in large quantities. The major constituents of this compound are cellulose, hemicellulose (hetero-polysaccharides including xylose, glucose, mannose, galactose, and arabinose), and lignin [4]. The carbon sources used in fermentative processes are important cost factors. Hence, lignocellulosic materials have attracted much attention as alternative carbon sources [5].

However, there are several technical limitations to the use of lignocellulose. Hydrolysis of lignocellulose produces a mixture of hexoses and pentoses, but most commercial lactic acid producer strains lack the ability to metabolize pentose [5]. The low lactic acid concentration and consequent inefficiency of the fermentation process presents another problem [6]. Polymeric materials must be broken down into fermentative sugars by enzymes or inorganic acids. When enzymatic hydrolysis and fermentation are performed sequentially, this is referred to as separate hydrolysis and fermentation (SHF). Enzymatic catalysis, involved in the hydrolysis step, can be inhibited by the sugars released. However, the two process steps can be performed simultaneously, in what is called simultaneous saccharification and fermentation (SSF) [3,7]. When both processes are carried out in the same reaction vessel, enzyme inhibition can be prevented, while the sugars released are rapidly converted by the microorganisms. SSF has the further advantages of lower equipment costs, lower contamination risk, shorter process time, and higher production rates [8]. The disadvantage of this type of biosynthesis is the fact that the conditions for enzymatic hydrolysis and fermentation have to be the same, so each process is carried out in a suboptimal environment [9].

Lactic acid is one of the primary platform chemicals, and can be used to synthesize a wide variety of useful products [10]. Lactic acid can participate in a wide variety of chemical reactions, including esterification, condensation, polymerization, substitution, or reduction [11]. Its reduced derivatives, such as propylene glycol, can be used as green chemicals in industrial applications [12]. Several methods of lactic acid biosynthesis have been investigated, including batch, fed-batch, semi-continuous/repeated batch, continuous fermentation, or SSF processes, using different lactic acid-producing strains, renewable starch (e.g., wheat starch, corn starch, sago starch, potato starch), or lignocellulose materials (e.g., woody materials, crop residues) [4]. Sugar beet pulp (SBP) has the potential to be a valuable substrate in various biotechnological processes. Cellulose, hemicellulose, and pectin constitute 60%–70% of its dry matter. Hydrolysis of these polymeric substances is a promising step towards valorizing this by-product from the sugar industry. The main constituent monosaccharides in sugar beet pulp are glucose, galacturonic acid, and arabinose (both present in pectin) [13].

Our previous research showed, for the first time, the possibility of using sugar beet pulp as a substrate for lactic acid fermentation [12]. The lactate obtained in this way was suitable for the synthesis of propylene glycol. The aim of the present research was to increase the efficiency of the process of lactic acid biosynthesis, through attenuation of the arabinose and galactose present in the fermented sugar beet pulp hydrolyzates after processes conducted with lactic acid bacteria (LAB) monocultures. We performed simultaneous saccharification and fermentation of sugar beet pulp using mixed cultures of LAB with different fermentations abilities.

## 2. Results and Discussion

### 2.1. Sugar Beet Pulp Hydrolyzate as a Carbon Source for Lactic Acid Fermentation

Generally, glucose is the best carbon source for lactic acid fermentation with bacterial strains. However, lactic acid can be produced efficiently and economically using lignocellulosic substrates, such as sugar beet pulp [4]. The dry basis of sugar beet pulp (a by-product from the sugar industry) is composed mainly of polysaccharides consisting of 22–24 wt% cellulose, 30 wt% hemicelluloses, and 15–25 wt% pectin, with small amounts of fat, protein, ash, and lignin at 1.4 wt%, 10.3 wt%, 3.7 wt%, and 5.9 wt%, respectively [14]. The carbohydrate composition of sugar beet pulp consists predominantly of glucose, in the form of cellulose, and xylose, glucose, mannose, galactose, and arabinose in the form of hemicelluloses. Sugar beet pectin (a polymeric galacturonic acid backbone with intermittent blocks of alternating monomers, unbranched galactan, and highly branched arabinan side chains) consists of glucose with arabinose and galacturonic acid, together with smaller amounts of galactose and rhamnose [15,16,17]. All of these building blocks were detected in our study during hydrolysis conducted with multienzyme preparations (Table 1). Lactic acid bacteria are able to assimilate most of the carbohydrates from the hydrolyzates. However, the concentration of specific carbohydrates is important to ensure efficient fermentation. Therefore, we tested three different types of enzymatic pretreatment, resulting in various compositions of carbohydrates in the hydrolyzate, to be used as a fermentation medium.

### 2.2. Carbon Utilization Profiles of Tested LAB Strains

Lactic acid bacteria ferment hexoses and pentoses via different metabolic pathways. Homo-fermentation produces lactic acid from hexose and pentose as the only end products, via the Embden-Meyerhof-Parnas (EMP) pathway and the pentose phosphate (PP) pathway, respectively. Despite the fact that glucose is the most favorable carbon source for most LAB, co-fermentation with mixed sugars has been recognized as an effective method of lactic acid fermentation in several experiments conducted using both wild-type and engineered strains [4]. The utilization profiles of all tested bacteria, both collection strains (PCM 2510; PCM 2379; PCM 490; PCM 488; ATCC 8014; PCM 2675) and environmental isolates (Hydr II; R; AXD; AXG), were measured in batch fermentations conducted in SHF mode. After enzymatic hydrolysis (stopped thermally), the carbohydrate composition of the sugar beet pulp was characterized and the ability of the bacteria to utilize the carbohydrates was assessed (Table 2). On the basis of their assimilation profiles, the tested LAB strains were divided into two types. The first group of strains utilized mostly hexoses (glucose, fructose, and mannose): *L. acidophilus* PCM 2510, *Lactococcus lactis* PCM 2379, *L. delbrueckii* PCM 490, *L. plantarum* H II, and *L. plantarum* R. Lower utilization rates were observed in strains from the second group: *L. brevis* PCM 488, *L. plantarum* ATCC 8014, *L. plantarum* PCM 2675, *L. plantarum* AXD, and *L. plantarum* AXG. Group II also showed a lesser ability to utilize arabinose. Whereas the constitutive enzymes for glucose fermentation are synthesized continually, the synthesis of enzymes specific for pentose assimilation and fermentation must be induced [4].

Most homofermentative strains, including *Lactobacillus delbrueckii* and *Lactobacillus acidophilus*, can produce lactic acid from glucose, but not hemicellulose-derived sugars, such as xylose or arabinose [18]. This fact was confirmed in our study (Table 2). Only some lactobacilli are able to grow on pentoses [19]. Some studies have reported that *L. plantarum* only co-metabolizes pentoses in complex media with yeast extracts [19,20]. Other studies, conducted by Gobbetti et al., have shown that cell growth with arabinose as a carbon source does not require special medium supplementation [20]. The consumption of raffinose, rhamnose, and xylose by the tested LAB differed, not depending on the group of strains (Table 2). Carbon sources, such as xylose, galactose, arabinose, and fructose derived from lignocellulose are less effective substrates for fermentation processes than glucose [2]. In particular, it can be difficult to achieve efficient fermentation using hydrolyzates of mixed sugars including pentoses and hexoses by monocultures [4]. To promote efficient fermentation we, therefore, used mixed populations of lactic acid bacteria, with various assimilation profiles.

### 2.3. Lactic Acid Fermentation of Sugar Beet Hydrolyzates Using Mixed Cultures

**Figure 1:**
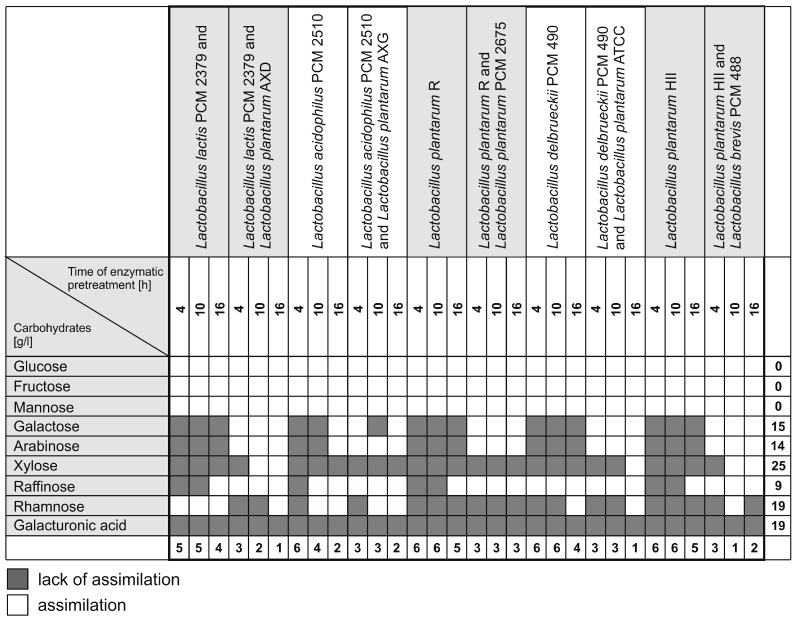
Assimilation profiles for the first and second steps of the SSF processes.

A number of difficulties stand in the way of effective utilization of lignocellulosic biomass for lactic acid production. These are mainly connected with the differentiation and complexity of the carbohydrate profiles. Efficient co-utilization of different sugars could reduce the production cost of bio-based chemicals. Only a few LAB strains have been reported to consume sugars derived from lignocelluloses simultaneously [13,21,22]. It was, therefore, decided to ferment sugar beet hydrolyzates using mixed cultures in SSF mode. Based on their carbohydrate utilization profiles, the LAB strains were divided into two groups with different abilities. Five pairs of strains were identified as being possibly complementary (Table 3). In mixed populations, these strains showed the potential to utilize a broader spectrum of sugars released from beet pulp than in monocultures. The fermentation trials were conducted in two stages. In the first step, sugar beet pulp carbohydrates, mainly glucose, were fermented with Group I monocultures. In the next step, the same trials were inoculated with the second strain of a particular pair, and unfermented galactose and arabinose were consumed by the mixed cultures. Figure 1 shows the patterns of assimilation profiles for the first (monocultures) and second steps (mixed culture) in the SSF processes.

Improvements in the effectiveness of both hydrolysis and fermentation were noticed in the second process step (Table 4). For the pair *Lactobacillus plantarum* HII and *Lactobacillus brevis* PCM 488, effectiveness expressed as the amount of hydrolyzate mass was 48.31 g after the first step (conducted in monocultures) and 58.36 g after the second step (mixed cultures). An increase in lactic acid productivity (from 55.79 to 59.57 g/L) was also observed.

### 2.4. Lactic Acid Fermentation of Sugar Beet Hydrolyzates in SSF Mode

Many types of biomass provide rich sources of carbohydrates, which can be converted biologically into lactic acid. Enzymatic hydrolysis, especially in SSF processes, can improve the efficiency of the subsequent fermentation process significantly. The effectiveness of SSF processes depends not only on the abilities of LAB, but also on the kind of substrate used, especially its carbohydrate content. In studies conducted by John et al., [23], a lactic acid yield around 40% higher than the average obtained in our study was achieved using mixed cultures of *L. casei* and *L. delbrueckii* for the fermentation of cassava bagasse hydrolyzates. Lactic acid production using corn stover as a carbon source was also substantially improved by inoculation with mixed cultures of *L. rhamnosus* and *L. brevis* [21]. The use of more than one fermenting strain can improve the effectiveness of the process by around 10%–30%. Lactic acid production from date juice was 53 g/L and 42 g/L using single cultures of *Lactobacillus casei* and *Lactococcus lactis*, respectively, while a mixed culture of the bacteria increased the yield to 60.3 g/L [24].

The purpose of enzymatic hydrolysis is to break down polysaccharides into easily fermentable sugars [4]. However, in the process the sugars generated may strongly inhibit enzyme activity (feedback inhibition), with the disadvantage, among others, of requiring more enzyme loads. SSF is, therefore, an effective solution, whereby enzymatic hydrolysis and fermentation can be conducted while the released sugars are being metabolized [4]. Simultaneous carbohydrate utilization reduces carbon catabolite repression, which is observed with most lactic acid bacteria, since glucose represses the consumption of other less-favorable sugars, such as xylose and arabinose [25]. However, according to the literature, some LAB strains are capable of utilizing hexoses and pentoses to produce lactic acid. For example *L. bifermentans* DSM 20003 can convert the mixture of glucose, arabinose, and xylose in wheat bran hydrolyzate, and *L. pentosus* ATCC 8041 can covert sugars released from renewed trimming vine shoots and corn cobs into lactic acid [18].

**Figure 2:**
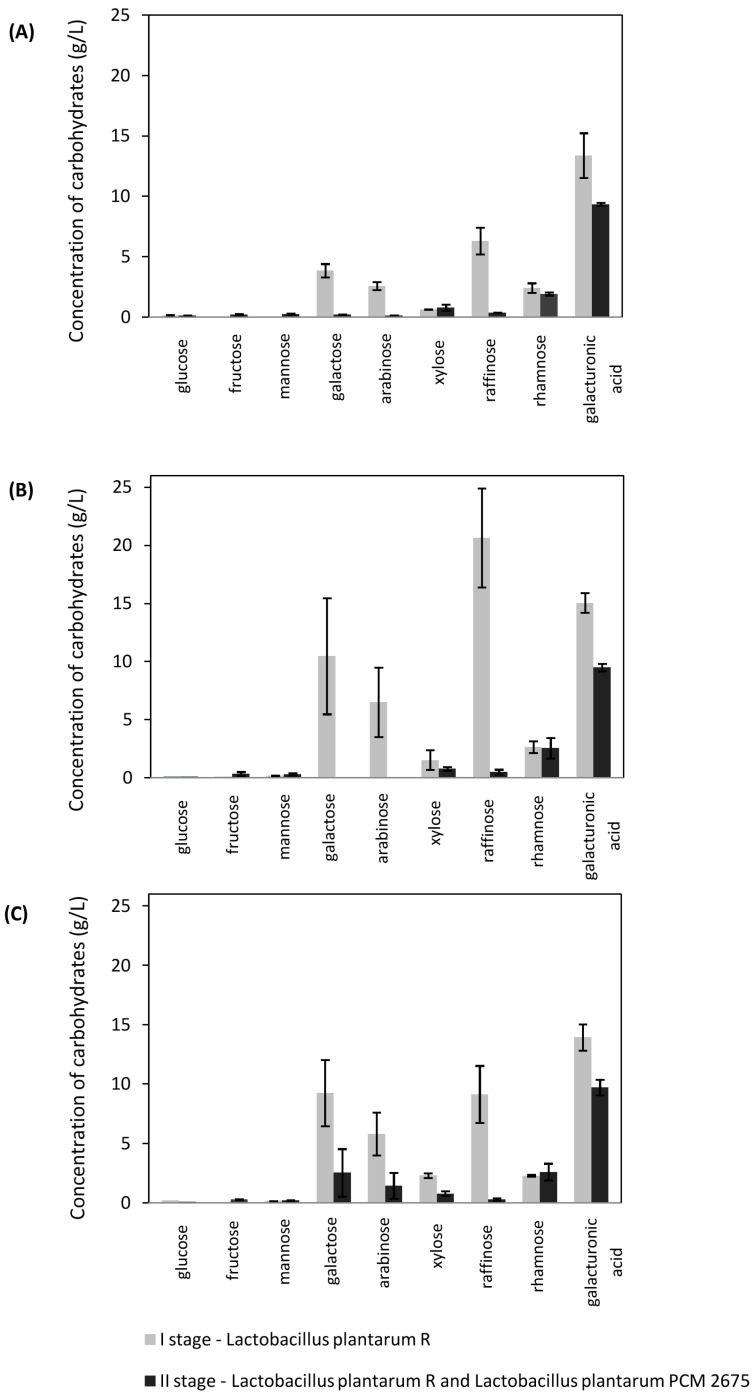
Concentration of carbohydrates (g/L) after (**A**) four hours, (**B**) 10 h, and (**C**) 16 h for I stage (*Lactobacillus plantarum* R) and II stage (*Lactobacillus plantarum* R and *Lactobacillus plantarum* PCM 2675).

**Figure 3:**
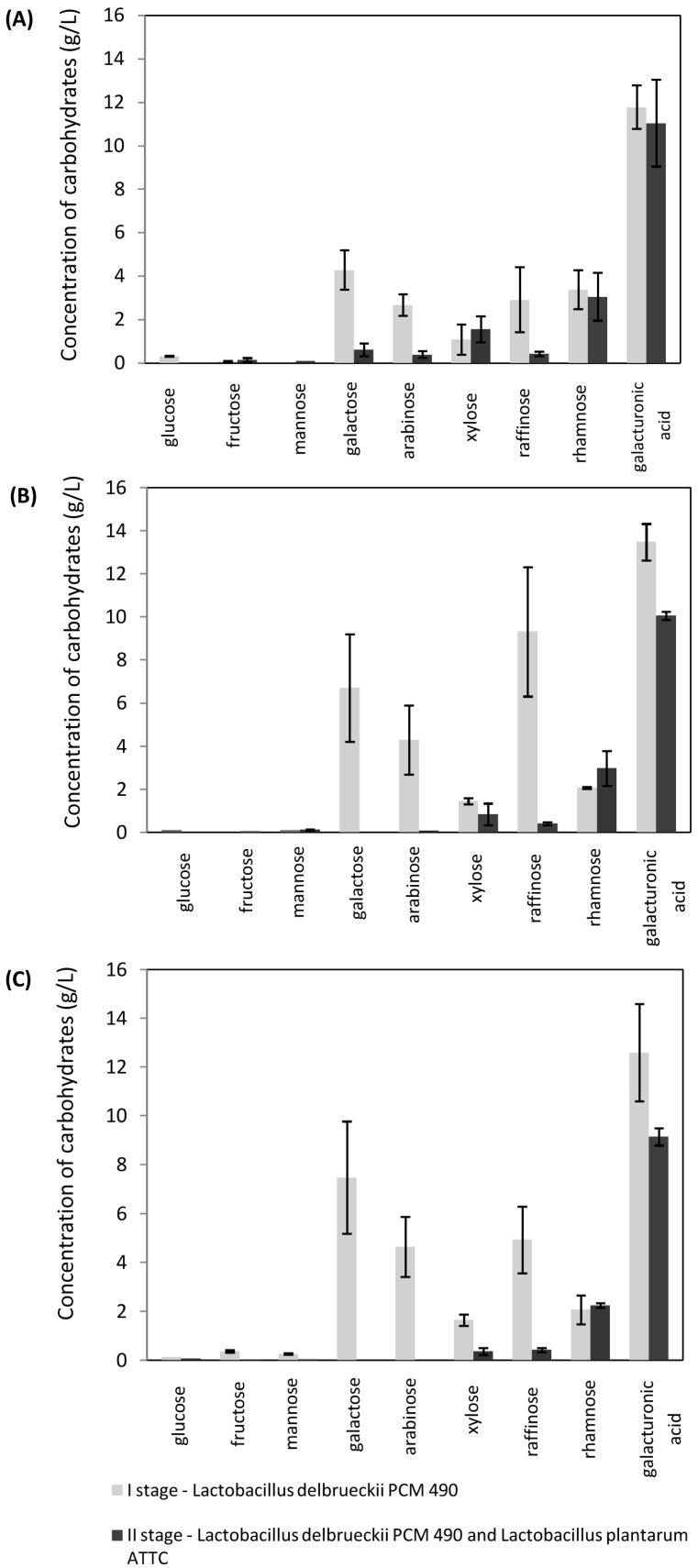
Concentration of carbohydrates (g/L) after (**A**) 4 h, (**B**) 10 h, and (**C**) 16 h of enzymatic pretreatment for I stage (*Lactobacillus delbrueckii* PCM 490) and II stage (*Lactobacillus delbrueckii* PCM 490 and *Lactobacillus plantarum* ATCC 8014).

In this study, arabinose and xylose were utilized in the second stage of the fermentation process, conducted using LAB mixed cultures. Analysis of carbohydrate contents (Figure 2 and Figure 3) indicates that the post-fermentation medium after the monoculture step did not contain glucose and the carbon sources consumed in this stage were mainly galactose, arabinose, and raffinose. In most cases, xylose was found to be partially utilized. Taniguchi [26] also reports that a co-culture of *Enterococcus casseliflavus* and *L. casei* produced lactic acid from xylose and glucose. It is notable that the xylose concentrations after the first step were higher than those prior to fermentation. This shows the progress of enzymatic hydrolysis in sugar beet pulp. According to our results, a marker for biomass degradation could be the concentration of galacturonic acid. Figure 2 shows the composition of the fermentation medium after *Lactobacillus plantarum* R and *Lactobacillus plantarum* PCM 2675 cultivation. Figure 3 presents results for the process conducted using *Lactobacillus delbrueckii* PCM 490 and *Lactobacillus plantarum* ATCC 8014 cultures. In both cases, the content of galacturonic acid in the hydrolyzate doubled within the first 48 h.

The initial culture conditions, such as pH, temperature, and inoculum size, were considered as important factors determining cell growth and lactic acid yield [4]. We also investigated the influence of initial carbohydrate concentrations on the effectiveness of the SSF process. Three varying courses of biomass enzymatic pretreatment were tested. The sugar profiles of the hydrolyzates obtained after four, ten and sixteen hours of enzymatic hydrolysis are shown in Table 1. We observed variations between the fermentation processes, depending on the pretreatment conditions. In all cases, the glucose and fructose released during both the pretreatment and SSF processes were metabolized completely, whereas galactose and arabinose concentrations increased after the first fermentation step, independently of the mode of pretreatment (Table 1, Figure 2 and Figure 3). It can be concluded that these sugars were not metabolized by monocultures in the first stage. This may have been due to carbon catabolite repression. This cellular regulation process occurs when microorganisms are exposed to two or more carbon sources and only one is preferentially utilized [27]. Catabolite repression is usually related to the glucose effect [28]. According to Ishola et al. [23], lowering the concentration of glucose in the fermentation environment may improve xylose uptake, facilitating simultaneous sugar utilization.

**Figure 4:**
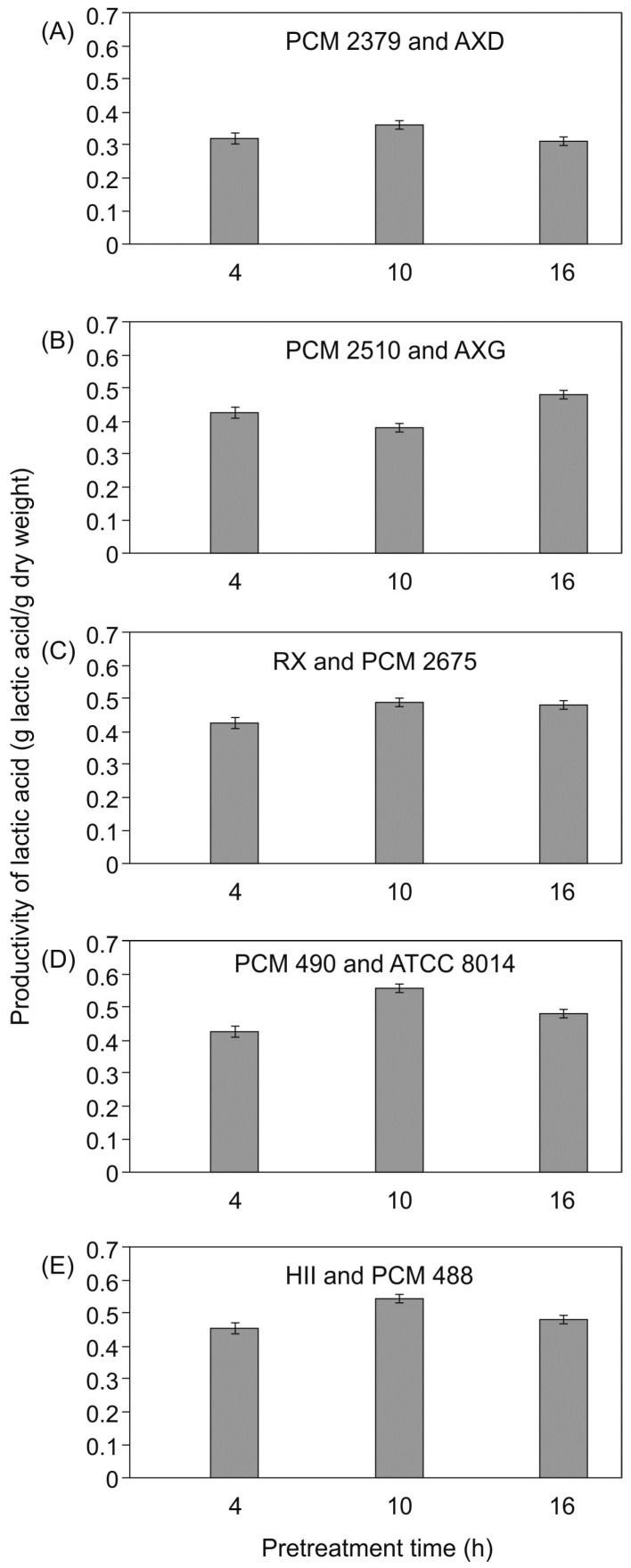
Productivity of lactic acid for (**A**) *Lactococcuss lactis* PCM 2379 and *Lactobacillus plantarum* AXD, (**B**) *Lactobacillus acidophilus* PCM 2510 and *Lactobacillus plantarum* AXG, (**C**) *Lactobacillus plantarum* R and *Lactobacillus plantarum* PCM 2675, (**D**) *Lactobacillus delbrueckii* PCM 490 and *Lactobacillus plantarum* ATCC 8014, and (**E**) *Lactobacillus plantarum* HII and *Lactobacillus brevis* PCM 480.

Siragusa [29] demonstrated that the capacity for metabolic adaptation of *L. plantarum* was dependent on the type of strain and cultivation media. In our research, four *L. plantarum* strains showed a variety of abilities to metabolize less favorable hydrolyzate carbohydrates in the absence of glucose. The amount of raffinose determined in post-fermentation hydrolyzates varied significantly with different strain pairs. However, the largest amounts of this sugar were measured after the first step of fermentation, in media inoculated after 10 h of enzymatic pre-hydrolysis (Figure 2 and Figure 3). Utilization of compounds remaining after the first process step led to increases in lactic acid concentrations in the second step (Table 4). The lactic acid yield per 1 g of sugar beet pulp dry weight was calculated, taking into consideration variations in the effectiveness of hydrolysis (Figure 4). With most cultures, the highest values were observed for processes initiated after 10 h of pretreatment. Values varied in the range from 0.39–0.55 g/g. Few papers have investigated lactic acid production from renewable materials using different fermentation methods [3,4,18]. Reported product yields range from 0.28 to 0.99 g lactic acid/g of substrate. The best results (>0.9) are reported to have been achieved with substrates such as brewer’s spent grain, molasses, wheat straw, apple pomace, or sago starch. Lower productivities (<0.4) have been observed for rice bran and sugarcane bagasse. Similar yields to those in our study were reported by John et al. [30] and Shen and Xia [31], when cassava bagasse or corn cob residues were fermented using *L. casei* NCIMB 3254 and *L. delbruecki* ZU-S2, respectively.

### 2.5. Biologically-Synthesized Lactic Acid as a Platform Chemical for Propylene Glycol Synthesis

Oxygenated chemicals, such as propylene glycol, propylene oxide, acrylic acid, and acrylate esters, or other chemical intermediates (including lactate ester plasticizers), can be made from lactic acid by way of catalytic transformations. Table 5 presents results obtained with the pair *Lactobacillus delbrueckii* PCM 490 and *Lactobacillus plantarum* ATCC, which showed the highest lactic acid productivity (see Figure 4). A water solution of lactic acid was used for the model system. In both cases, catalytic reduction over 5% Ru/C led to the formation of propylene glycol as the main product. Moreover, increased selectivity towards this product was obtained with higher concentrations of lactic ions in the reaction mixture.

The lactic acid solution was biologically synthesized for catalytic studies. The concentrations of lactate ions obtained varied between 41.80–57.64 g/L, in the same range as the water solution of commercial lactic acid used in our investigations. The same tendency was observed in the catalytic reactions of biologically-prepared samples, as in the case of pure reagents. The rise in the initial concentration of lactate ions led to increased selectivity to propylene glycol.

## 3. Materials and Methods

### 3.1. Biological Material

#### 3.1.1. Bacterial Strains

Six collection strains (Polish Collection of Microorganisms PCM) were used: *Lactococcus lactis* PCM 2379, *Lactobacillus plantarum* PCM 2675, *Lactobacillus acidophilus* PCM 2510, *Lactobacillus brevis* PCM 488, *Lactobacillus delbrueckii* PCM 490, *Lactobacillus plantarum* ATCC 8014; and four environmental isolates from self-fermented grass and sugar beet: *Lactobacillus plantarum* HII, *Lactobacillus plantarum* R, *Lactobacillus plantarum* AXD, *Lactobacillus plantarum* AXG. Environmental isolates were identified using molecular methods based on 16S rRNA gene sequencing and deposited in the NCBI GenBank database with the following accession numbers: KT751284 (*Lactobacillus plantarum* AXD), KT751285 (*Lactobacillus plantarum* AXG), KT751286 (*Lactobacillus plantarum* HII), KT751287 (*Lactobacillus plantarum* R).

#### 3.1.2. Plant Material

Sugar beet pulp (18% of dry weight) was obtained from a sugar factory in Dobrzelin, Poland. Sugar beet pulp was suspended in plain warm water to achieve a dry matter concentration of around 12% (*w/v*). The biomass was saccharified in a 5 L reactor for 24 h at 37 °C using a mixture (1:1) of two multi-enzyme preparations made by Novozymes: Viscozyme® and Ultraflo® Max (Bagsvaerd, Denmark) (0.03 L/kg of sugar beet pulp dry weight). The resulting medium contained glucose (12.56 g/L), fructose (5.11 g/L), xylose (0.48 g/L), rhamnose (2.44 g/L), raffinose (21.72 g/L), galactose (16.36 g/L), and arabinose (9.63 g/L). Saccharification was stopped by heating (80 °C for 10 min).

### 3.2. Hydrolysis and Fermentation

#### 3.2.1. Inoculum Preparation

Cultures of LAB were grown in glass tubes for 48 h at 37 °C. Each strain was passaged six times using sugar beet pulp hydrolyzate. Saccharified hydrolyzate was used for the propagation of LAB strains after supplementation with non-sugar de Man, Rogosa, and Sharpe (MRS) ingredients (BTL, Lodz, Poland) containing: yeast extract, beef extract, peptone K, triammonium citrate, dipotassium hydrogen phosphate, sodium acetate, magnesium sulfate × 7 H_2_O, manganese sulfate × 4 H_2_O. Followed doses per sugar beet pulp dry mass were added: yeast extract 0.16%, beef extract 0.33%, peptone K 0.42%, triammonium citrate 0.08%, dipotassium hydrogen phosphate 0.08%, sodium acetate 0.2%, magnesium sulfate × 7 H_2_O 0.08%, manganese sulfate × 4 H_2_O 0.002%. The medium was sterilized at 120 °C and stored in a refrigerator.

#### 3.2.2. Sugar Beet Pulp Biomass Preparation and Enzymatic Pre-Treatment

The fermentations were conducted in 100 mL Erlenmeyer flasks filled with 50 mL of medium. Sugar beet pulp was suspended in plain warm water to achieve a dry matter concentration of around 12% (*w/v*) and after sterilization a mixture was added of two commercial multienzyme preparations: 0.1 mL of Viscozyme (Novozymes, Bagsvaerd, Denmark)) and 0.1 mL Ultraflo Max (Novozymes)/50 mL medium. Calcium carbonate (CaCO_3_; Avantor Performance Materials Poland S.A., Gliwice, Poland) was used as a neutralizing agent for pH stabilization. Hydrolysis was conducted at 37 °C for 4, 10, and 16 h. The sugar beet pulp hydrolyzate was supplemented with mineral and nitrogen compounds (yeast extract, beef extract, peptone K, triammonium citrate, dipotassium hydrogen phosphate, sodium acetate, magnesium sulfate × 7 H_2_O, manganese sulfate × 4 H_2_O; see point 3.2.1).

#### 3.2.3. Fermentation

**Figure 5:**
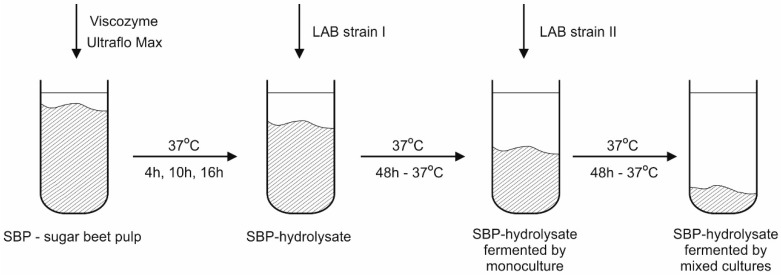
Simultaneous saccharification and lactic acid fermentation of sugar beet pulp.

Fermentations with LAB monocultures (I stage of inoculation) and mixed cultures (II stage of inoculation), using the strain pairs presented in Table 3, were conducted at 37 °C for 48 h in hydrolyzate media without deactivation of hydrolytic enzymes. The entire procedure is presented in Figure 5. After 4, 10, or 16 h of pre-hydrolysis, 5 mL of inoculum (first strain) was added. After 48 h of incubation, the samples were inoculated using a second strain, while the pH was maintained at 5 by the addition of sterile 80% CaCO_3_ (Avantor Performance Materials Poland S.A., Gliwice, Poland). After another 48 h of incubation at 37 °C, the process was halted by heating (80 °C for 10 min), which deactivated the enzymes and increased the solubility of the calcium lactate.

#### 3.2.4. Effectiveness of Hydrolysis

To compare the progress of hydrolysis in each step of the SSF process, the mass of the hydrolyzate obtained was measured after separation of the solid fraction (centrifugation, 10 min, 3000× *g*).

### 3.3. Propylene Glycol Synthesis

Biological lactic acid samples were acidified to pH 2.5 using H_2_SO_4_ (96%) and purified on active carbon (ERCARBON GE , Erbslöh Geisenheim AG, Geisenheim, Germany, 2.5 g/50 mL). The solid adsorbent was weighed and added to a metered volume of a liquid biological sample. The mixture was stirred vigorously for one minute at 10 °C. The supernatant was then separated by filtration and subjected to catalytic hydrogenation over 5% Ru/C (Sigma-Aldrich, Saint Louis, MO, USA, CAS 206180) [32]. Hydrogenation of lactic acid was performed in a 50 mL autoclave (Parr Instrument Company, Moline, IL, USA) at 130 °C and under 3.5 MPa of H_2_ pressure. The reactions were conducted with equal amounts of catalyst (m_cat_ = 0.5 g). The mixture was stirred at 500 rpm. The autoclave was flushed with Ar, then flushed again with H_2_ and pressurized with H_2_ to 3.5 MPa. The temperature was gradually raised to 130 °C at a heating rate of 20 °C·min^−1^. The reaction was sustained for four hours. After the reaction, the autoclave was cooled to room temperature and the reaction mixture was filtered and analyzed using HPLC and GC-FID. Selectivity for propylene glycol was determined using the equation:

(1) $$S=\left[\frac{C_{PG}}{\left(C_{0}-C\right)}\right]\times 100\%$$

where *C*_PG_ is the concentration of propylene glycol (M), *C*_0_ is the initial concentration of lactate ions [M] and *C* is the concentration of lactate ions at time t (M).

### 3.4. Culture Media Analysis

The efficiency of sugar beet pulp hydrolysis was measured as hydrolyzate weight (g) after hydrolysis and separation of solid residues (centrifugation, 3000× *g*, 10 min). The monosaccharide profile of the sugar from the beet hydrolyzates was analyzed using UV spectrophotometry and Megazyme (Wicklow, Ireland) kits. For glucose, mannose, and fructose, we used the d-Mannose, d-Fructose, and d-Glucose assay kit (K-MANGL); for arabinose, the l-Arabinose and d-Galactose assay kit (K-ARGA); for galacturonic acid, the d-Glucuronic Acid and d-Galacturonic Acid assay kit (K-URONIC); for xylose, the d-Xylose assay kit (K-XYLOSE); for raffinose, the Raffinose/d-Galactose assay kit (K-RAFGA); for rhamnose, the l-Rhamnose assay kit (K-RHAMNOSE). The concentration of lactate ions was measured using spectrophotometry and a d-/l-Lactic Acid assay kit (K-DLATE) (Megazyme, Wicklow, Ireland).

## 4. Conclusions

Processes involving the transformation of lactic acid into green chemicals could compete with conventional chemical processes if the efficiency and cost effectiveness of the process of lactic acid production were improved. The result of this study was the increase of the efficiency of the process of lactic acid biosynthesis from fermented sugar beet pulp hydrolyzates. A series of controlled batch fermentations was conducted under various conditions, including pretreatment with enzymatic hydrolysis. Simultaneous consumption of less favorable carbon sources (galactose, arabinose, and raffinose) by lactic acid bacteria was observed in mixed cultures with complementary assimilation abilities. The pattern of sequential inoculation, as well as the selection of LAB pairs with complementary assimilation patterns, was an important factor in the processes, leading to increased lactic acid yield. The propylene glycol obtained from sugar bio-synthesized lactic acid was as pure as that obtained from an aqueous solution of lactic acid. Over 95% selectivity was achieved for both commercial lactic acid and biological samples with lactate ion concentrations above 50 g/L.

## Figures and Tables

**Table 1:** Composition of the sugar beet pulp medium during hydrolysis (0.1 mL of Viscozyme and 0.1 mL Ultraflo Max (Novozymes)/50 mL).

Time of hydrolysis	Carbohydrate Concentration (g/L)		
	4 h	10 h	16 h
Glucose	18.61 ± 0.70	21.79 ± 0.54	29.74 ± 1.19
Fructose	4.52 ± 0.40	8.90 ± 0.29	12.46 ± 0.60
Mannose	3.04 ± 0.14	5.97 ± 0.17	7.04 ± 0.45
Arabinose	1.54 ± 0.50	2.60 ± 0.87	3.47 ± 0.82
Galactose	2.27 ± 0.90	3.90 ± 0.39	5.18 ± 0.31
Raffinose	2.48 ± 0.59	8.61 ± 0.71	16.24 ± 0.60
Rhamnose	0.88 ± 0.59	1.75 ± 0.08	2.26 ± 0.30
Xylose	0.39 ± 0.053	0.48 ± 0.038	0.47 ± 0.049
Galacturonic acid	3.66 ± 0.24	5.51 ± 0.44	7.81 ± 0.19

**Table 2:** Carbohydrate utilization profiles of lactic acid bacteria after fermentation of sugar beet pulp hydrolyzates (medium obtained acc. to 3.1.2.).

Strain	Compound Utilization (%)								
	**Glucocse**	**Fructose**	**Mannose**	**Arabinose**	**Galactose**	**Raffinose**	**Rhamnose**	**Xylose**	**Galacturonic acid**
*Lactobacillus plantarum* Hydr II	100	99	94	0	0	35	7	0	37
*Lactobacillus acidophilus* PCM 2510	100	100	95	0	0	0	0	0	27
*Lactococcus lactis* PCM 2379	100	100	99	0	0	0	0	0	37
*Lactobacillus delbrueckii* PCM 490	100	100	90	0	0	29	33	0	57
*Lactobacillus plantarum R*	100	99	91	0	0	42	7	0	37
*Lactobacillus plantarum* AXD	73	100	77	7	0	13	0	0	45
*Lactobacillus plantarum* AXG	68	100	78	26	0	0	0	0	35
*Lactobacillus brevis* PCM 488	67	55	82	10	0	29	4	0	35
*Lactobacillus plantarum* ATCC 8014	73	100	84	9	0	16	0	0	42
*Lactobacillus plantarum* PCM 2675	76	100	81	5	7	11	0	0	59

**Table 3:** Pairs of LAB strains used for fermentation.

LAB Strains	Group I of LAB Strains	Group II of LAB Strains
I pair	*Lactococcuss lactis* PCM 2379	*Lactobacillus plantarum* AXD
II pair	*Lactobacillus acidophilus* PCM 2510	*Lactobacillus plantarum* AXG
III pair	*Lactobacillus plantarum* HII	*Lactobacillus brevis* PCM 488
IV pair	*Lactobacillus delbrueckii* PCM 490	*Lactobacillus plantarum* ATCC 8014
V pair	*Lactobacillus plantarum* R	*Lactobacillus plantarum* PCM 2675

**Table 4:** Effectiveness of hydrolysis and lactic acid fermentation of sugar beet pulp in SSF processes conducted with monocultures (step I) and mixed cultures (step II). Experiment started after 10 h of enzymatic pretreatment.

Strains	Step	Hydrolyzate (g)	Lactic Acid (g/L)
*Lactococcus lactis* PCM 2379	I	44.79 ± 0.81	47.88 ± 1.50
*Lactococcus lactis* PCM 2379 *and Lactobacillus plantarum* AXD	II	46.85 ± 0.04	49.66 ± 0.70
*Lactobacillus acidophilus* PCM 2510	I	53.74 ± 1.08	42.60 ± 2.37
*Lactobacillus acidophilus* PCM 2510 *and Lactobacillus plantarum* AXG	II	55.66 ± 0.10	52.61 ± 1.65
*Lactobacillus delbrueckii* PCM 490	I	49.90 ± 0.11	57.78 ± 0.26
*Lactobacillus delbrueckii* PCM 490 and *Lactobacillus plantarum* ATCC	II	57.06 ± 0.97	58.39 ± 1.40
*Lactobacillus plantarum* R	I	46.92 ± 0.40	51.34 ± 0.26
*Lactobacillus plantarum* R and *Lactobacillus plantarum* PCM 2675	II	57.37 ± 0.62	53.75 ± 0.14
*Lactobacillus plantarum* HII	I	48.31 ± 0.10	55.79 ± 0.54
*Lactobacillus plantarum* HII and *Lactobacillus brevis* PCM 488	II	58.36 ± 0.48	59.57 ± 0.83

**Table 5:** Activity and selectivity of 5% Ru/C catalyst during reduction of lactate ions to propylene glycol.

Substratum	Initial Concentration of Lactate Ions (g/L)	Conversion of Lactate Ions (%)	Selectivity to Propylene Glycol (%)
Water solution of lactic acid	45.04	96.41	63.12
	60.32	52.71	96.82
Biologically synthesized lactic acid solution *	41.80	53.63	67.42
	43.96	88.76	68.44
	44.32	51.11	83.43
	45.85	49.52	94.12
	47.11	38.24	96.89
	50.08	43.83	94.89
	57.64	40.64	97.37

* Purified and acidified post-fermentation sugar beet pulp hydrolyzates obtained using *Lactobacillus delbrueckii* PCM 490 and *Lactobacillus plantarum* ATCC. Samples were diluted with water to different concentrations.
